# A retrospective survey of strabismus surgery in a tertiary eye center in northern China, 2014–2019

**DOI:** 10.1186/s12886-021-01805-w

**Published:** 2021-01-14

**Authors:** Xiaomei Wan, Luqin Wan, Mingming Jiang, Yichao Ding, Yuan Wang, Jing Zhang

**Affiliations:** 1 Qingdao Eye Hospital of Shandong First Medical University, No. 5 Yan’er Dao Road, Shinan District, 266071 Qingdao, Shandong China; 2 State Key Laboratory Cultivation Base, Shandong Provincial Key Laboratory of Ophthalmology, Shandong Eye Institute, Shandong First Medical University& Shandong Academy of Medical Sciences, No. 5 Yan’er Dao Road, Shinan District, 266071 Qingdao, Shandong China; 3 Medical College of Qingdao University, Qingdao, 266000 China

## Abstract

**Background:**

To evaluate the distribution pattern and changes of strabismus surgery in northern China.

**Methods:**

The records of strabismus patients at Qingdao Eye Hospital from January 2014 to December 2019 were reviewed retrospectively. The characteristics analyzed included gender, regional distribution, constituent ratio of age and type of strabismus. Changes during the periods 2014–2016 and 2017–2019 were compared and analyzed.

**Results:**

A total of 5746 strabismus patients were recruited. The number of strabismus patients was relatively stable each year from 2014 to 2016 but gradually increased each year from 2017 to 2019. Of these, 51.7% (2968/5746) were male, and 48.3% (2778/5746) were female. The majority (89.8%, 5159/5746) of the patients were from Shandong Province. The statistical results of the constituent ratio of age showed that 32.4% (1860/5746) were 7–12 years old (primary school level). Patients under 12 years of age (preschool and primary school level) accounted for 60.0% (3447/5746) of all the patients. In terms of the types of strabismus, exotropia accounted for 63.5% (3650/5746), followed by esotropia and vertical rotational strabismus at 13.2% (758/5746) and 9.7% (555/5746), respectively. Intermittent exotropia was the most common type among the exotropia patients, accounting for 71.3% (2604/3650). Among the patients with intermittent exotropia, 62.5% (1627/2604) were children aged 4–12 years, and the basic type of intermittent exotropia was the main type. Four percent (231/5746) of the patients, of which adult patients comprised the main population, required reoperation.

**Conclusions:**

Patients with strabismus at primary school level comprised the largest group of strabismus patients in north China. Exotropia was the most common type of strabismus, and intermittent exotropia was the most common type of exotropia. The rate of exotropia to esotropia was 5:1.

## Background

Epidemiologic studies of strabismus focus predominantly on Western countries. In Asian countries, they are mainly concentrated in Singapore and Japan. However, due to differences in ethnicity, population size, and regional distribution, the findings of these studies can only provide reference for clinicians in China and cannot really guide their clinical work. In China, because of differences in diagnostic and treatment levels, many hospitals do not carry out strabismus surgery or carry out fewer strabismus surgeries. It is therefore difficult to obtain data on strabismus surgery distribution without a large sample. For this study, a retrospective analysis of strabismus patients during a 6-year period in a tertiary eye center in north China was conducted to understand the regional distribution and constituent ratios of age and types of strabismus. This study therefore sought to determine the distribution of strabismus in a tertiary eye center in northern China and to provide a reference for the distribution pattern of strabismus surgery in China.

## Methods

The data of 5746 patients who underwent strabismus surgery in the Strabismus Department of Qingdao Eye Hospital from January 2014 to December 2019 were collected retrospectively. All the clinical records were provided by the information centre system of Qingdao Eye Hospital. This information included each patient’s name, gender, age, address, and diagnosis, among other details. In each case, the naked and corrected binocular vision, intraocular pressure, refractive state, anterior segment, and ocular fundus were examined prior to surgery. The 6 m and 33 cm degrees of horizontal or vertical deviation were measured using the Hirschberg method and prism cover test. The deviation degrees of the other directions and binocular visual function were checked according to the needs of the surgery. The diagnosis and classification of strabismus were based on expert consensus (Strabismus and Pediatric Ophthalmology Group of the Ophthalmology Branch of the Chinese Medical Association, 2015) on strabismus classification in China [1]. Indications for surgery: The surgical criteria for exotropia included a degree of deviation (distance and/or near vision) ≧ 20 PD, a frequency of deviation occurring for more than 1/2 of all waking hours, and/or these effects combined with a sensory deficit. The surgical criteria for esotropia were mainly based on the effect of deviation when wearing corrective glasses in patients with a continued deviation (distance and/or near vision) ≧ 15PD with the corrective glasses. The surgical criteria for vertical strabismus included a degree of deviation ≧ 10PD combined with a compensatory head position. SPSS version 22.0 statistical software was used to analyze the data in this retrospective study. The enumeration data were compared using a chi-squared (*X*^2^) test, and *P* < 0.05 was considered statistically significant.

The study was approved by the Ophthalmology Ethics Committee of Qingdao Eye Hospital (approval no.2020-21). Written informed consent was obtained from all the patients who participated in the study or their legal representatives. Written consent was obtained from a parent or guardian on behalf of any participants under the age of 16 before the study.

## Results

### **Pattern distribution in strabismus surgery** p**atients**

**Fig. 1:**
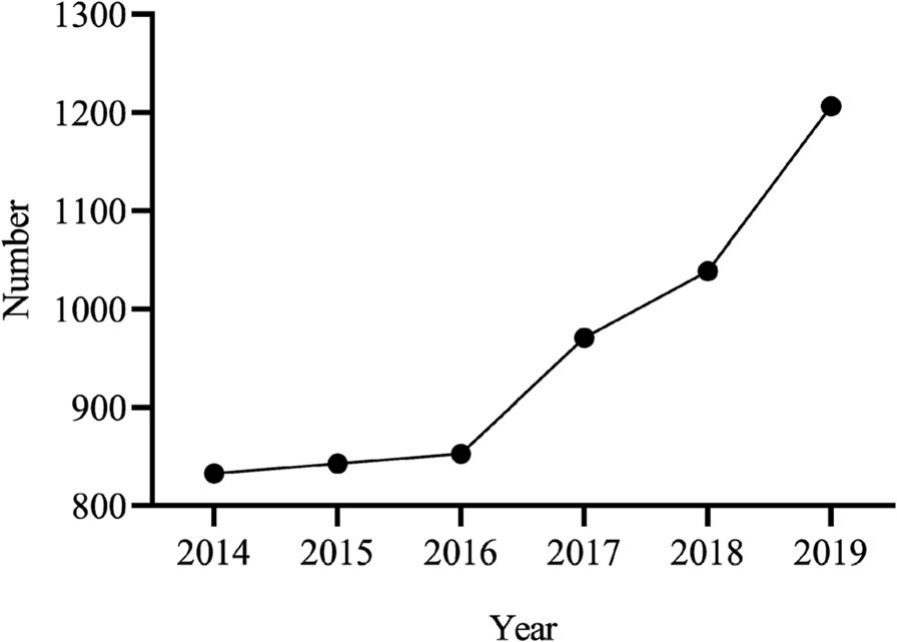
Changes in trends among the strabismus patients at Qingdao Eye Hospital: 2014–2019

The data of 5746 strabismus patients who visited Qingdao Eye Hospital from January 2014 to December 2019 were recorded and analysed. The patients’ ages ranged from 1 to 84 years, with an average age of 15.2 years. The horizontal strabismus angle was 0–160 PD, and the vertical deviation was 0–90 PD. Among the patients, 6.3% (364/5746) had an anterior segment abnormality, and 2.3% (130/5746) had a posterior segment abnormality. The number of strabismus patients was relatively stable each year from 2014 to 2016 (833, 843, 853, respectively) but gradually increased each year from 2017 to 2019 (971, 1039, 1207, respectively) (Fig. 1). In terms of gender, 51.7% (2968/5746) were male, and 48.3% (2778/5746) were female. There was no significant difference between the gender composition over time (*P =* 0.146) (Table 1).

**Table 1:** Characteristics of the strabismus patients at Qingdao Eye Hospital: 2014–2019

Selected characteristics	2014–2016	2017–2019	Total	*P*
	N(%)	N(%)	N(%)	
**Total**	2529	3217	5746	
**Area**				
In Qing	1226(48.5)	1504(46.8)	2730(47.5)	
Outside Qingdao	1046(41.4)	1383(43.0)	2429(42.3)	0.408
Outside Shandong	257(10.2)	330(10.3)	587(10.2)	
**Gender**				
Male	1279(50.6)	1689(52.5)	2968(51.7)	0.146
Female	1250(49.4)	1528(47.5)	2778(48.3)	
**Age**				
0–6	683(27.0)	904(28.1)	1587(27.6)	0.021
7–12	790(31.2)	1070(33.3)	1860(32.4)	
13–18	300(11.9)	400(12.4)	700(12.2)	
>18	756(29.9)	843(26.2)	1599(27.8)	
**Type**				
Esotropia	286(11.3)	472(14.7)	758(13.2)	
Exotropia	1689(66.8)	1961(61.0)	3650(63.5)	
A-V strabismus	99(3.9)	137(4.3)	236(4.1)	
Vertical rotational strabismus	234(9.3)	321(10.0)	555(9.7)	0.000
Special types of strabismus	135(5.3)	225(7.0)	360(6.3)	
Central paralytic strabismus	42(1.7)	33(1.0)	75(1.3)	
Nystagmus	44(1.7)	68(2.1)	112(1.9)	

The strabismus patients resided in three areas: within Qingdao, outside Qingdao (within Shandong Province) and outside Shandong Province. The statistical results over the 6-year study period showed that the majority of the patients were from Shandong Province: 47.5% (2730/5746) of the patients were from Qingdao, 42.3% (2429/5746) were from other cities in Shandong Province and 10.2% were from other provinces (587/5746) (Table 1).

The patients were divided into four age groups: 0–6 years (preschool level), 7–12 years (primary school level), 13–18 years (middle school level), and over 18 years (adults). The statistical results of the constituent ratio of age showed that 9.3% (534/5746) of the patients were 0–3 years old, 27.6% (1587/5746) were 0–6 years old, 32.4% (1860/5746) were 7–12 years old, 12.2% (700/5746) were 13–18 years old, and 27.8% (1599/5746) were over 18. There were significant differences between the age groups over time (*P* = 0.021). Among the patients with strabismus, the 7–12-year-olds (primary school level) comprised the largest group, followed by those who were over 18 (adults). The patients under 12 years (preschool and primary school level) accounted for 60.0% (3447/5746) of all the strabismus patients (Table 1).

All the categorizations were based on the classification of strabismus formulated by the Strabismus and Pediatric Ophthalmology Group of the Ophthalmology Branch of Chinese Medical Association. Among the patients, the majority (63.5%, 3650/5746) had exotropia, followed by esotropia and vertical rotational strabismus at 13.2% (758/5746) and 9.7% (555/5746), respectively. Intermittent exotropia was the most common type of exotropia, accounting for 71.3% (2604/3650) of patients. Special types of strabismus made up 6.3% (360/5746) of the patient population. Among these, the most common type was dissociated vertical deviation (DVD), which accounted for 87.5% (315/360). A-V strabismus, nystagmus and central paralytic strabismus comprised a small proportion of the patients in this study at 4.1% (236/5746), 1.9% (112/5746) and 1.3% (75/5746), respectively. Among all the patients, the exotropia group was the largest, and intermittent exotropia was the most common type of exotropia. The differences in the types of strabismus became statistically significant as time passed (*P* = 0.000). The rate of esotropia was 11.3% (286/2529) from 2014 to 2016 and increased to 14.7% (472/3217) during 2017–2019. Meanwhile, the rate of exotropia was 66.8% (1689/2529) from 2014 to 2016 but decreased to 61.0% (1961/3217) during the period 2017–2019 (Table 1).

### Pattern distribution of intermittent exotropia

Intermittent exotropia was the most common type of exotropia in this study, so the gender composition, constituent ratio of age and types of strabismus of the 2604 intermittent exotropia cases were analyzed statistically. The results showed that 50.8% (1322/2604) of the patients were male, and 49.2% (1282/2604) were female. There was no significant difference between the two groups in terms of gender (*P* = 0.054). The statistical results of the constituent ratio of age showed that 29.8% (775/2604) of the patients were 0–6 years of age. Among them, 10.2% (265/2604) were under 3 years of age, 19.6% (510/2604) were 4–6 years old, 42.9% (1117/2604) were 7–12 years old, 12.4% (322/2604) were 13–18 years old, and 15.0% (390/2604) were over 18. There were no significant differences between the different age groups as time passed (*P* = 0.303). Among the patients with strabismus, the primary school level patients (7–12 years old) comprised the largest group, followed by the preschool level patients (0–6 years old). Meanwhile, patients under 12 years of age (preschool and primary school level) accounted for 72.7% (1892/2604) of all patients, among whom 62.5% (1627/2604) were 4–12 years old. Intermittent exotropia was classified into the basic type, the convergence insufficiency type and the divergence-excess type, and these accounted for 79.8% (2077/2604), 12.1% (314/2604) and 8.2% (213/2604), respectively, of the intermittent exotropia patients in the study, with the basic type thus the most prevalent form. The differences between the three types over the 6-year study period were statistically significant over time (*P* = 0.004) (Table 2).

**Table 2:** Characteristics of the intermittent exotropia patients at Qingdao Eye Hospital: 2014–2019

Selected characteristics	2014–2016	2017–2019	Total	*P*
	N(%)	N(%)	N(%)	
**Total**	1058	1546	2604	
**Gender**				
Male	513(48.5)	809(52.3)	1322(50.8)	0.054
Female	545(51.5)	737(47.7)	1282(49.2)	
**Age**				
0–6	326(30.8)	449(29.0)	775(29.8)	0.303
7–12	457(43.2)	660(42.7)	1117(42.9)	
13–18	133(12.6)	189(12.2)	322(12.4)	
>18	142(13.4)	248(16.0)	390(15.0)	
**Type**				
Basic type	874(82.6)	1203(77.8)	2077(79.8)	
Convergence insufficiency type	102(9.6)	212(13.7)	314(12.1)	0.004
Divergence-excess type	82(7.8)	131(8.5)	213(8.2)	

### Pattern distribution of strabismus patients undergoing reoperation

Four percent (231/5746) of all the patients required reoperation. Among them, 43.7% (101/231) were male, and 56.3% (130/231) were female. The majority, 88.3% (204/231), were from Shandong Province. The proportion of patients aged over 18 was 54.5% (126/231), which was the highest percentage among all the age groups. On the other hand, the proportion of patients aged 0–6 years was 3.5% (8/231), which was the lowest proportion among all the age groups. Adult patients were the main population group who underwent reoperation (Table 3).

**Table 3:** Characteristics of the patients undergoing reoperation at Qingdao Eye Hospital: 2014–2019

Selected characteristics	2014–2019
	N(%)
**Total**	231
**Gender**	
Male	101(43.7)
Female	130(56.3)
**Area**	
in Shandong	204(88.3)
Out Shandong	27(11.7)
**Age**	
0–6	8(3.5)
7–12	60(26.0)
13–18	37(16.0)
>18	126(54.5)

### Characterisation of strabismus classification at different ages

**Fig. 2:**
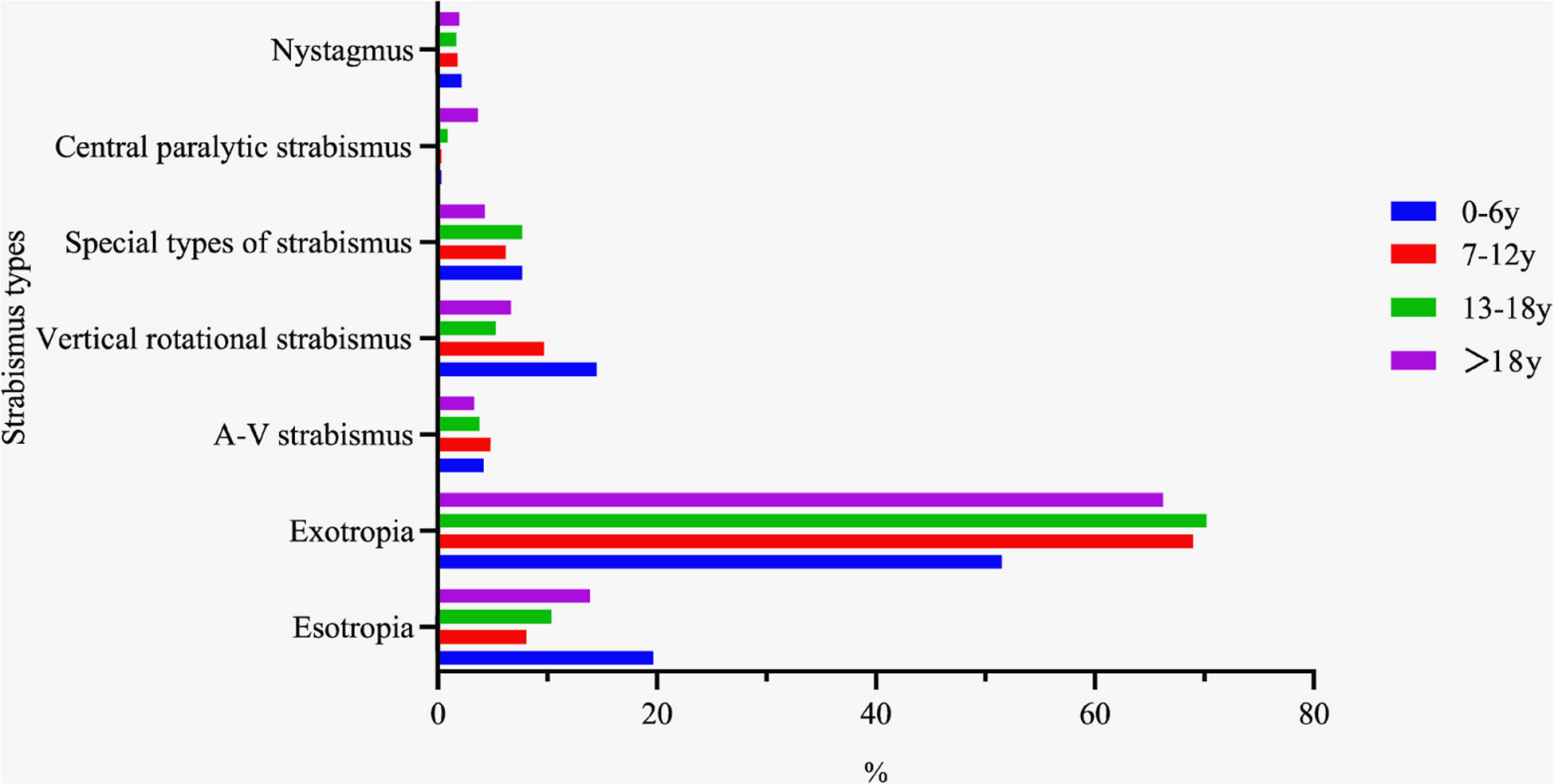
Strabismus classification of patients of different ages at Qingdao Eye Hospital: 2014–2019

We analysed the ratios of the different types of strabismus among the four age groups. Esotropia was highest among patients aged 0–6 years (19.7%, 312/1587), while exotropia was lowest among patients aged 0–6 years (51.5%, 817/1587). The highest proportion of patients with vertical rotatory strabismus was those aged 0–6 years at 14.5% (230/1587). We calculated the proportion of patients with congenital strabismus in the group aged from 0 to 6 years. Among them, the proportion of congenital esotropia was 8.5% (135/1587), whereas the proportion of congenital exotropia was 9.2% (147/1587). The highest proportion of patients with central paralytic strabismus comprised those older than 18 years (3.7%, 59/1597), while the lowest proportions were patients aged 0–6 years and 7–12 years at 0.3% (4/1587 and 6/1860, respectively). The differences in the types of strabismus were statistically significant as time passed among the patients aged 0–6 years and older than 18 years (*P* = 0.000; *P* = 0.001, respectively). The rates of esotropia for these groups were 16.8% (115/683) and 10.6% (80/755), respectively, during the period 2014–2016 and increased to 21.8% (197/904) and 16.9% (142/842), respectively, from 2017 to 2019. Among the patients aged 0–6 years and older than 18 years, the rates of exotropia were 58.3% (398/683) and 68.9% (520/755), respectively, from 2014 to 2016, but these decreased to 46.3% (419/904) and 63.8% (537/842), respectively, during the 2017–2019 period. There was no statistical difference over time among the patients aged 7–12 years and 13–18 years (*P* = 0.471; *P* = 0.485, respectively) (Table 4; Fig. 2).

**Table 4:** Characterisation of the strabismus classification of patients of different ages at Qingdao Eye Hospital: 2014–2019

Selected characteristics	2014–2016	2017–2019	Total	*P*
	N(%)	N(%)	N(%)	
**Total**	2529	3217	5746	
**0–6**				
Esotropia	115(16.8)	197(21.8)	312(19.7)	
Exotropia	398(58.3)	419(46.3)	817(51.5)	
A-V strabismus	20(2.9)	47(5.2)	67(4.2)	
Vertical rotational strabismus	98(14.3)	132(14.6)	230(14.5)	0.000
Special types of strabismus	36(5.3)	86(9.5)	122(7.7)	
Central paralytic strabismus	2(0.3)	2(0.2)	4(0.3)	
Nystagmus	14(2.0)	21(2.3)	35(2.2)	
**7–12**				
Esotropia	65(8.2)	86(8.0)	151(8.1)	
Exotropia	557(70.5)	726(67.9)	1283(69.0)	
A-V strabismus	40(5.1)	50(4.7)	90(4.8)	
Vertical rotational strabismus	73(9.2)	108(10.1)	181(9.7)	0.471
Special types of strabismus	38(4.8)	78(7.3)	116(6.2)	
Central paralytic strabismus	3(0.4)	3(0.3)	6(0.3)	
Nystagmus	14(1.8)	19(1.8)	33(1.8)	
**13–18**				
Esotropia	26(8.6)	47(11.7)	73(10.4)	
Exotropia	214(71.1)	279(66.9)	493(70.2)	
A-V strabismus	15(5.0)	12(3.0)	27(3.8)	
Vertical rotational strabismus	15(5.0)	22(5.5)	37(5.3)	0.485
Special types of strabismus	21(7.0)	33(8.2)	54(7.7)	
Central paralytic strabismus	4(1.3)	2(0.5)	6(0.9)	
Nystagmus	6(2.0)	6(1.5)	12(1.7)	
**>18**				
Esotropia	80(10.6)	142(16.9)	222(13.9)	
Exotropia	520(68.9)	537(63.8)	1057(66.2)	
A-V strabismus	24(3.2)	28(3.3)	52(3.3)	
Vertical rotational strabismus	48(6.4)	59(7.0)	107(6.7)	0.001
Special types of strabismus	40(5.3)	28(3.0)	68(4.3)	
Central paralytic strabismus	33(4.4)	26(3.1)	59(3.7)	
Nystagmus	10(1.3)	22(2.6)	32(2.0)

### Clinical risk factors in patients 0–12 years old with exotropia and esotropia

The majority of the patients were under 12 years of age. Among the patients, the majority had exotropia and esotropia, so we further analyzed the personal and family histories of the strabismus patients under 12 years of age. We also analyzed the correlation between the type of strabismus and the refractive state. The rate of premature delivery, oxygen inhalation, and the family history of strabismus and eye diseases was higher in patients with esotropia (5.4%, 25/463; 3.9%, 18/463; 9.1%, 42/463; 7.8%, 36/463) than with exotropia (3.6%, 75/2100; 1.2%, 25/2100; 7.7, 162/2100; 3.9%, 82/2100). Additionally, 62.4% (289/463) of the patients with esotropia showed hyperopia, while 7.3% (154/2100) of the patients with exotropia showed hyperopia in refractive status. By contrast, 5.2% (24/463) of the patients with esotropia showed myopia, while 39.9% (838/2100) of the patients with exotropia showed myopia in refractive status. The rate of emmetropia was significantly higher in patients with exotropia (28.0%, 587/2100) than with esotropia (3.7%, 17/463) (Table 5).

**Table 5:** Clinical risk factors in patients aged 0–12 years with exotropia and esotropia at Qingdao Eye Hospital: 2014–2019

Risk factor	Exotropia	Esotropia
	N(%)	N(%)
**Total**	2100	463
**History of premature delivery**	75(3.6)	25(5.4)
**History of oxygen inhalation**	25(1.2)	18(3.9)
**Family history of strabismus**	162(7.7)	42(9.1)
**Eye disease**	82(3.9)	36(7.8)
**Refractive state**		
Emmetropia	587(28.0)	17(3.7)
Myopia	838(39.9)	24(5.2)
Hyperopia	154(7.3)	289(62.4)
Astigmatism	306(14.6)	95(20.5)
Anisometropia	215(10.2)	38(8.2)

## Discussion

The results of epidemiological studies of strabismus have shown that the prevalence of strabismus among different populations varies by region and ethnicity. The prevalence of strabismus among white and African American children from 6 to 71 months was 3.3% and 2.1%, respectively. Esotropia and exotropia accounted for half the strabismus cases [2]. These were 3.55% and 3.24%, respectively, among Asian and non-Hispanic white children. Strabismus was found to be higher in children aged 6–72 months [3]. In Singapore, the prevalence of strabismus among children aged 6–72 months was found to be 0.80%, and the ratio of exotropia to esotropia was 7:1, with 63% of exotropia being intermittent [4]. Among 2704 patients with horizontal strabismus in Hong Kong, exotropia was shown to be more common than esotropia. In addition, the proportion of patients with intermittent exotropia seemed to be increasing [5]. Meanwhile, paralytic strabismus was the most common strabismus among adults [6]. Esotropia is most common strabismus in the first 10 years of life, and a population-based study found that accommodative and acquired nonaccommodative forms of childhood esotropia occur most frequently [7]. Intermittent exotropia and insufficient convergence are the most common forms of childhood exotropia [8]. The results of this study showed that exotropia accounted for the highest proportion of all strabismus cases at 63.5%, followed by esotropia at 13.2%. The ratio of exotropia to esotropia was thus 5:1. There are several reasons that could explain this result: (1) The incidence of different types of strabismus may be related to race, genetics and refractive errors. It has been reported that prematurity and maternal smoking during pregnancy are associated with a higher risk of pediatric esotropia and exotropia [9]. Furthermore, strabismus was associated with gestational age, hyperopic refractive error, and astigmatism [10]. Esotropia, with lower gestational age and a heavier placenta; exotropia, with a maternal history of previously treated hypertension and maternal use of recreational drugs during early pregnancy [11]. (2) Early screening of children’s vision and binocular visual function is associated with the incidence of exotropia. Early vision screening provides an opportunity for intermittent exotropia to be detected, which increases the diagnostic levels of the disease. (3) The choice of surgery timing on the basis of the characteristics of the disease could play an important role. Most children with esotropia have refractive errors and amblyopia. Both early precision optics and improved eye adjustment function have a corrective effect on the eye position in esotropia, which can reduce the need for surgical intervention. Contrarily, surgery is the primary treatment for patients with exotropia. Early strabismus surgery could not only reduce the damage to binocular visual function caused by strabismus, but also facilitate the establishment of visual function after surgery.

Intermittent exotropia is the most common type of exotropia, accounting for 50–90% of all patients with exotropia [12]. The results of this study showed that intermittent exotropia makes up the highest proportion (71.3%) of all patients with exotropia. Furthermore, exotropia constitutes the highest proportion of all strabismus patients. It can be speculated that surgery for intermittent exotropia is currently the main type of strabismus surgery. In this study, the main surgical group of intermittent exotropia patients comprised preschool and primary school-aged children, and the basic type was the main type of intermittent exotropia. The number of strabismus patients was relatively stable each year from 2014 to 2016, but gradually increased annually over the 2017–2019 period. This reason for this can be explained as follows: Many patients or the parents of children understand and receive early strabismus treatment because of a concomitant improvement in the national quality of life and awareness of strabismus. Adult patients were the main population of patients who underwent reoperation, which may be related to the high recurrence rate of strabismus over time. Esotropia was the highest form of strabismus and exotropia the lowest in patients aged 0–6 years, and this may be related to esotropia often being associated with hyperopic refractive abnormalities. Furthermore, exotropia may be related to the occurrence and development of myopia.

It was found that patients with intermittent exotropia are at a great higher risk of visual fatigue in comparison with healthy individuals [13]. Strabismus patients showed a greater risk of developing thoracic scoliosis [14].The temporal integration for stereopsis is impaired in patients with IXT, requiring longer critical integration time to achieve elevated optimal stereoacuity[15].Children with untreated strabismus can develop impaired binocular vision, which can interfere with their ability to conduct social interactions with other children. Lack of binocularity and stereopsis in children is associated with significant motor skills impairment, in particular for static balance and catching tasks [16]. Children can subsequently develop a sense of inferiority and fail to lead normal lives. Both the child and their parents’ health-related quality of life showed a trend toward correlating with clinical severity [17, 18]. Strabismus surgery has a positive impact on children’s physical and psychological functioning. Children with greater corrections experience greater improvements in their quality of life after surgery [19]. The stereopsis and health-related quality of life in adults with childhood large angle exotropia can be improved after successful surgical correction [20]. At the same time, with improvements in the national quality of life and awareness of children’s diseases, many parents of children with strabismus understand and accept that early treatment of strabismus can enable children to obtain good stereo vision. Importantly, populations with strabismus need early treatment. This study showed that, among the patients who underwent strabismus surgery, those at primary school level (i.e., 7–12 years old) comprised the largest group, while those at preschool and primary school level (i.e., aged 0–12 years) accounted for 60% of all patients who underwent strabismus surgery. The parents of children in the latter age group should choose early strabismus surgery. In addition to being concerned that strabismus may cause damage to binocular visual function, some parents also worry about the impact of strabismus on the normal psychological development of their children.

With improvements in living standards, the prevention and treatment of strabismus and amblyopia have gradually been carried out in various parts of China, and emphasis has been placed on children’s diseases and the need for early interventions. Many children with strabismus in relatively economically developed areas are screened and treated in hospitals. In addition, with the continuous improvements in ophthalmologists’ technical levels, the treatment of strabismus has become more professional, which has increased the probability of successful surgery. In areas with relatively less-advanced medical standards where there are a lack of professional ophthalmologists, some ophthalmologists have relatively little knowledge of strabismus or do not pay enough attention to the disease. They believe that strabismus surgery should be provided when the child is older, thus giving patients the wrong information and causing problems. These children miss the optimal time for surgery, impairing their binocular vision and/or missing the best age to establish binocular vision. At the same time, because of the economic prosperity in China, excessive medical treatment of strabismus and amblyopia has become a prominent, negative medical phenomenon. Excessive medical treatment of strabismus includes violations of treatment standards and expanded operative indications. Accordingly, every ophthalmologist should master the standardized operative indications for strabismus. Promotional efforts and education about strabismus should not only be carried out in hospitals, but also in communities and schools so that every citizen can acquire a certain level of knowledge of strabismus. At the same time, ophthalmologists should perform eye examinations on preschool children so that children with strabismus can be detected early.

This research collected data exclusively from patients who underwent strabismus surgery in a tertiary eye center in northern China. However, many strabismus patients, especially those with mild intermittent exotropia, can be managed with non-surgical methods. A limitation of the present study is that it is a retrospective case analysis, rather than a population-based study. Since all samples were collected in the same hospital, the overall population of cases of strabismus is not described; therefore, we cannot know the incidence of strabismus surgery in the region. The geographical distribution was relatively narrow, as most of the patients were from Shandong Province, so this study was limited to some extent, and the results cannot be generalized. Further population-based studies are needed to confirm the overall incidence of strabismus and the proportion of strabismus surgery in China.

## Conclusions

The data analysis showed that primary school children comprised the largest strabismus group in northern China. Exotropia was the most common type of strabismus, and intermittent exotropia was the most common type of exotropia. Furthermore, the rate of exotropia to esotropia was 5:1. Strategies for strabismus should aim to educate children and their parents about strabismus, as well as the importance of early screening and interventions.

## Data Availability

The datasets used and/or analyzed during the current study are available from the corresponding author on reasonable request.

## Abbreviations

DVD: Dissociated vertical deviation
IXT: Intermittent exotropia
